# An automated images-to-graphs framework for high resolution connectomics

**DOI:** 10.3389/fninf.2015.00020

**Published:** 2015-08-13

**Authors:** William R. Gray Roncal, Dean M. Kleissas, Joshua T. Vogelstein, Priya Manavalan, Kunal Lillaney, Michael Pekala, Randal Burns, R. Jacob Vogelstein, Carey E. Priebe, Mark A. Chevillet, Gregory D. Hager

**Affiliations:** ^1^Department of Computer Science, Johns Hopkins UniversityBaltimore, MD, USA; ^2^Applied Physics Laboratory, Research and Exploratory Development Department, Johns Hopkins UniversityLaurel, MD, USA; ^3^Department of Biomedical Engineering, Johns Hopkins UniversityBaltimore, MD, USA; ^4^Institute for Computational Medicine, Johns Hopkins UniversityBaltimore, MD, USA; ^5^Department of Applied Math and Statistics, Johns Hopkins UniversityBaltimore, MD, USA

**Keywords:** pipeline, framework, connectomics, graph error, computer vision, images to graphs, big data, electron microscopy

## Abstract

Reconstructing a map of neuronal connectivity is a critical challenge in contemporary neuroscience. Recent advances in high-throughput serial section electron microscopy (EM) have produced massive 3D image volumes of nanoscale brain tissue for the first time. The resolution of EM allows for individual neurons and their synaptic connections to be directly observed. Recovering neuronal networks by manually tracing each neuronal process at this scale is unmanageable, and therefore researchers are developing automated image processing modules. Thus far, state-of-the-art algorithms focus only on the solution to a particular task (e.g., neuron segmentation or synapse identification). In this manuscript we present the first fully-automated images-to-graphs pipeline (i.e., a pipeline that begins with an imaged volume of neural tissue and produces a brain graph without any human interaction). To evaluate overall performance and select the best parameters and methods, we also develop a metric to assess the quality of the output graphs. We evaluate a set of algorithms and parameters, searching possible operating points to identify the best available brain graph for our assessment metric. Finally, we deploy a reference end-to-end version of the pipeline on a large, publicly available data set. This provides a baseline result and framework for community analysis and future algorithm development and testing. All code and data derivatives have been made publicly available in support of eventually unlocking new biofidelic computational primitives and understanding of neuropathologies.

## 1. Introduction

Brain tissue volumes imaged using electron microscopy today already contain many thousands of cells that can be resolved at the scale of a single synapse. The amount of information is daunting: in just 1 mm^3^ of brain tissue, we expect petabytes of data containing 10^5^ neurons and 10^9^ synapses (Braitenberg and Schüz, 1991). While this region is very small in physical volume compared to an entire brain, it is roughly the scale of a cortical column, a hypothetical fundamental organizing structure in the cortex (Mountcastle, 1957).

**Figure 1:**
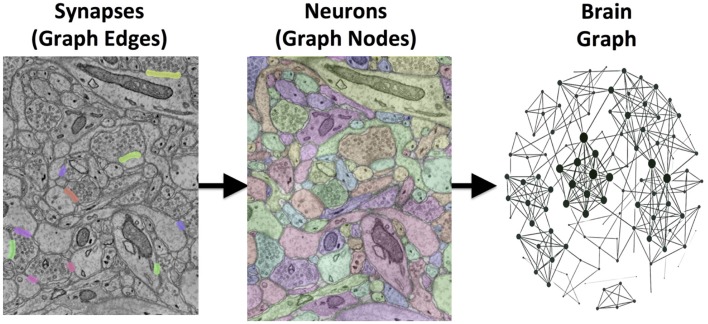
**An illustration of the images-to-graphs process**. Detected synapses are superimposed on raw EM data (left); these are overlaid and combined with multicolored neuron segments (middle) to estimate a graph (right). Nodes are represented by neurons and edges by synapses. The data shown here are a subset from a small, hand-labeled region of brain tissue. The graph (right) therefore represents a gold-standard brain network from this region of tissue using a standard graph layout (not corresponding to spatial position).

Our goal is to transform large 3D electron microscopy volumes of neural tissue into a detailed connectivity map, called a connectome. This approach will directly estimate brain graphs at an unprecedented level of detail. Each neuron is represented in the graph as a node, and each synapse is represented as an edge connecting these nodes (Figure 1). Manual human annotation, while currently the most accurate method of reconstruction, is unrealistic as volumes scale. A recent study estimated that manual annotation of a cortical column requires hundreds of thousands of person-years (Helmstaedter et al., 2011).

Therefore, an automated method to run algorithms at scale is needed to realize the promise of large-scale brain maps. We developed a novel ecosystem of algorithms, software tools and web services to accomplish this task.

We also introduce a fully-automated images-to-graphs pipeline and an assessment metric for the resulting graphs. This metric allows us to directly assess the connectivity properties of the graph, rather than relying on intermediate measures (e.g., synapse precision-recall or segmentation pixel error). We ran a grid search over a collection of parameters (i.e., both individual modules and their settings) using our pipeline to determine the best available result for analysis and interpretation. Once this optimal operating point was determined, we estimated the brain graph for a volume of neural tissue in our scalable framework. We believe that existing error metrics are inadequate to assess the overall quality of estimated graphs in high resolution connectomics data. Assessing graph error directly, as a system level evaluation (rather than component assessment), provides an improvement in the state of the art.

### 1.1. Previous work

Previous research methods have advanced the field of connectomics in important ways, but none have provided an end-to-end, automated, scalable approach. Several manual or semi-automated approaches have been used to construct brain circuits (Mishchenko et al., 2010; Bock et al., 2011; Takemura et al., 2013). Other groups have produced automated algorithms (Sommer et al., 2011; Nunez-Iglesias et al., 2013; Kaynig et al., 2015) that solve important pieces of the overall puzzle (e.g., neuron segmentation, synapse detection). These modules have generally been evaluated on small subvolumes without considering the overall graph result; additional work is needed to improve both algorithm accuracy and scalability for large graph inference.

In building our images-to-graphs pipeline, we leveraged previous work whenever available. To detect cell membranes, we reimplemented the ISBI 2012 challenge-winning approach (Ciresan et al., 2012), which framed membrane detection as a per-pixel classification problem and obtained state-of-the-art results using a small ensemble of Deep Neural Networks (DNN). We segment the neuronal structures by incorporating Rhoana (Kaynig et al., 2015), an open-source algorithm, which selects and fuses candidate 2D contours into 3D objects using conditional random fields (CRFs) and Integer Linear Programming (ILP). We also integrate Gala (Nunez-Iglesias et al., 2013), an agglomerative approach that combines super pixels into neurons. Together these two methods represent the two major approaches currently used in neuron segmentation; other methods can be readily adapted to this framework if desired. Scalable synapse detection (i.e., the edges in our graph) is still an open problem. While partial solutions exist (Becker et al., 2013; Kreshuk et al., 2014), they were developed for specific imaging paradigms (e.g., isotropic, post-stained). Therefore, we developed our own lightweight, scalable synapse detector. Finally, the Open Connectome Project (Burns et al., 2013) provides a high-performance spatial database optimized for processing large neuroimaging volumes. These tools facilitate scalable processing and provide significant advances over a flat-file architecture in terms of data storage and access.

## 2. Methods

This manuscript describes both our overall framework and infrastructure and also our images-to-graphs pipeline.

### 2.1. Framework

Our tools are built on a distributed processing framework which leverages the LONI Pipeline (Rex et al., 2003) for workflow management, and interfaces with the data and annotation services provided by the Open Connectome Project (OCP) (openconnecto.me). We provide an application programming interface (API) which implements our data standard for high resolution connectomics, and tools to enable rapid algorithm development while simplifying the challenge of running at scale. Our framework enables researchers to focus on developing novel image processing techniques without considering data management and processing challenges.

We are able to efficiently incorporate new methods by extracting only core algorithm code (often a small fraction of the original code base). We reuse our data management framework, eliminating the need for researchers to rewrite solutions for file handling and bookkeeping.

#### 2.1.1. RAMON

An acknowledged challenge in the connectomics field is annotation representation and its impact on software and institution-level interoperability (Lichtman et al., 2014; Plaza et al., 2014). As the field grows and data volumes increase, the sharing of data through remote and programmatic interfaces and the application of community developed algorithms and software will become common.

Answering this challenge requires scene parsing, rather than simply segmentation: the rich semantic annotations are critical to inferring graph structure and understanding the function and structure of neuronal circuits. We developed a standard for annotation metadata, as summarized in Table 1, which we call the Reusable Annotation Markup for Open coNnectomes (RAMON).

**Table 1:** **An overview of the current RAMON object types**.

**Type**	**Description**
SYNAPSE	Junction between two NEURONs is used to connect SEGMENTs when building a GRAPH
ORGANELLE	Represents internal cell structures (e.g., mitochondria, vesicles)
SEGMENT	A labeled region of a neuron; typically a contiguous voxel set
NEURON	Container for assembling SEGMENTs
NODE	Sparse annotation format for tracing processes or objects
SKELETON	An (organized) collection of NODEs, often used to represent a NEURON or arbor
ROI	An attributed region of interest, often used for atlases and other collections of labels
VOLUME	Used to store pixel label information; inherited by many other types
GENERIC	Extensible, used to specify arbitrary, user-defined information for a voxel set

*Each object is used to provide labels and attributes to objects identified in the neural tissue. This facilitates interoperability and efficient data storage, retrieval, and query processing*.

RAMON defines a minimum set of annotation types and associated metadata that capture important biological information and build the relationships between annotations that are necessary for connectome generation. Annotation metadata is trivially extensible through custom, user defined key-value pairs. This is not a formal ontology; instead it facilitates the development of software and tools by providing a flexible, yet reliable, standard for representing annotation data. For example, our synapse annotation type has fields such as weight, type, confidence, associated neural segments, and is extensible with arbitrary, searchable key-value pairs.

#### 2.1.2. Open connectome project (OCP)

Our approach for creating brain graphs from large image volumes (images-to-graphs) leverages the Open Connectome Project (www.openconnecto.me) infrastructure (Burns et al., 2013).

The Open Connectome Project (OCP) is a distributed, scalable data cluster, designed for spatial analysis and annotation of high-resolution brain data. It supports data from multiple imaging modalities (e.g., electron microscopy, light microscopy, array tomography, time series) with the goal of providing a unified backend for connectomics research. The system is designed using NoSQL data-stores and data-intensive architectures. The spatial index is partitioned, thus distributing the data across all the data-nodes to maximize throughput and avoid I/O interference.

OCP implements RAMON and provides publicly-accessible RESTful (REpresentational State Transfer) web-services that enable efficient data storage, retrieval and queries for images-to-graphs. This infrastructure provides the capability to efficiently view arbitrary data planes and access data subvolumes. Our pipelines store the output of computer vision algorithms as a set of queryable label and metadata annotations in an OCP spatial database co-registered with the image dataset. OCP provides a unified interface and backend for connectomics, and stores more than 70 unique data sets and thousands of annotation projects. These data sets constitute 50 TB on disk after compression, which is equivalent to more than 100 TB of raw data.

#### 2.1.3. Application programming interface

To enable rapid software development, we have created Connectome Annotation for Joint Analysis of Large data (CAJAL), an open source MATLAB Application Programming Interface (API), which implements the RAMON standard. The API provides classes for all RAMON annotation types, allowing users to easily develop interoperable algorithms and analysis tools. In addition to storing label data and metadata, the RAMON classes provide additional functionality such as tracking the annotation's global database location and visualizing a region of interest.

The API also provides an easy-to-use interface to query, upload, and download data from OCP services that abstracts complicated RESTful URL generation from the user. Many data formats are supported for both image and annotation data, including grayscale and multi-channel image data and integer and floating point based annotations. The toolbox automatically handles compression, chunking, and batching of annotation data to optimize throughput and simplify software development.

#### 2.1.4. Processing infrastructure

Data and results were stored using the Open Connectome Project. Our CPU compute cluster for images-to-graphs evaluation and deployment had a peak usage of 100 cores and 1 TB RAM. The overall cluster configuration consists of AMD Opteron 6348 cores (2.8 GHz, 12-core/processor, 4 processors/node) and 256 GB RAM per node. For membrane detection, we use a small GPU cluster containing 27 GeForce GTX Titan cards with 6 GB RAM. We leverage Son of Grid Engine (SGE) for task scheduling and the LONI Pipeline for workflow management (Rex et al., 2003). We parallelize at a data block level; each task is embarassingly parallel, and so we use traditional scheduling methods.

#### 2.1.5. Open connectome project services

**Figure 2:**
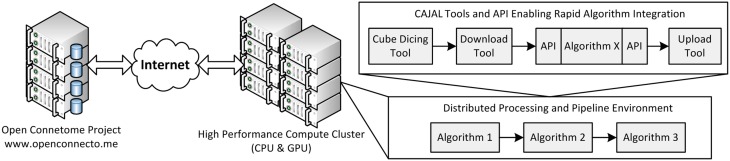
**An overall view of the framework, illustrating a distributed processing framework**. Data and annotation stores leverage the Open Connectome Project, and interface with a high perforamnce compute cluster. A variety of tools are available to facilitate a distributed processing environment.

OCP uses a load-balancing webserver (2x Intel Xeon X5650 2.67 GHz 12 core/processor and 48 GB of RAM). This webserver distributes jobs across three data servers running a distributed database (each with 2x Intel Xeon X5690 3.47 GHz 12 core/processor and 48 GB of RAM). Additionally, 100 TB of network mounted disk-space is available for storage (Burns et al., 2013). An overall schematic of our infrastructure is shown in Figure 2.

#### 2.1.6. Distributed block processing

**Figure 3:**
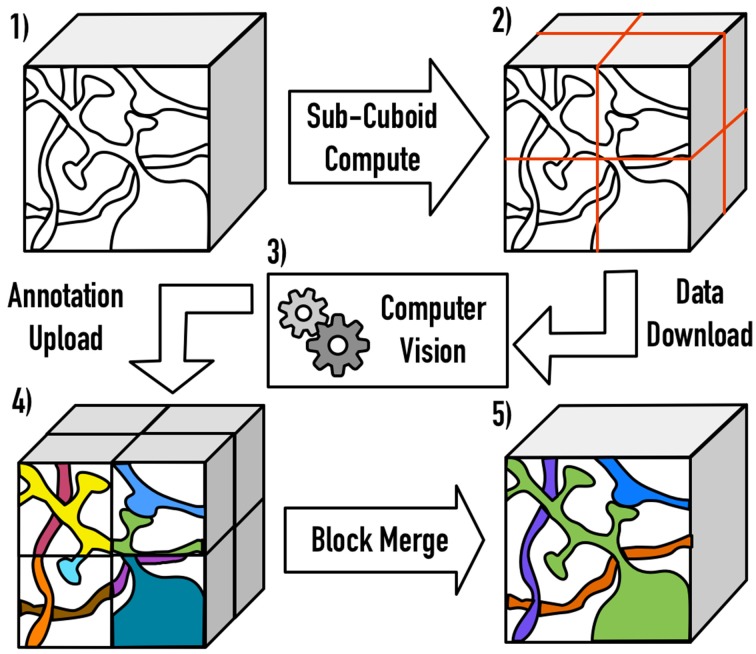
**An illustration of the distributed processing paradigm**. Raw image data (1) is divided into cuboids (2) based on user-specified parameters, with the necessary padding to perform computation. After processing (3), the annotations are inscribed and uploaded to OCP (4). Finally, processes are merged across block seams (5), using a similarity metric of the userś choice.

One major open challenge (e.g., in a file-based image system) is scaling Vision algorithms to massive volumes. We resolve this by extracting cuboids from large data volumes, accounting for overlapped and inscribed regions, processing the block, and then merging the annotations across the resulting cuboid boundaries. The current implementation serially processes large block seams, eliminating the need for transitive closure operations. As volumes scale, a hierarchical approach may be desirable to increase throughput (Figure 3).

### 2.2. Images-to-graphs pipeline

Often computer vision and other analytic tools for connectomics are written targeting a specific dataset, analysis product, or research question, and are only run on a limited data volume. Also, researchers invest much time in developing the required support software to manage data and facilitate processing. This results in code that is challenging to leverage and results that can be difficult to share or reproduce. To leverage state of the art ideas across the connectomics community, a flexible and robust system is beneficial to integrate and evaluate disparate tools, especially when scaling processing to massive data volumes.

We aimed to build a framework for connectomics processing that was agnostic to the underlying algorithms and provided reusable modules for common steps such as volume dicing, image data download, annotation upload, and annotation merging. By leveraging RAMON, Open Connectome Project, our API, and the LONI Pipeline workflow manager (Rex et al., 2003), we built a system capable of rapidly integrating, running, and evaluating connecomics algorithms on a single workstation or on a high performance compute cluster.

#### 2.2.1. Assessment measures

A variety of error measures have been proposed for connectomics (e.g., warping error, adjusted Rand index, variation of information, Nunez-Iglesias et al., 2013), but are limited by their focus on an individual subtask of the entire images-to-graphs pipeline. As we demonstrate, the optimal results for a subtask may not translate to optimal results for the overall pipeline.

**Figure 4:**
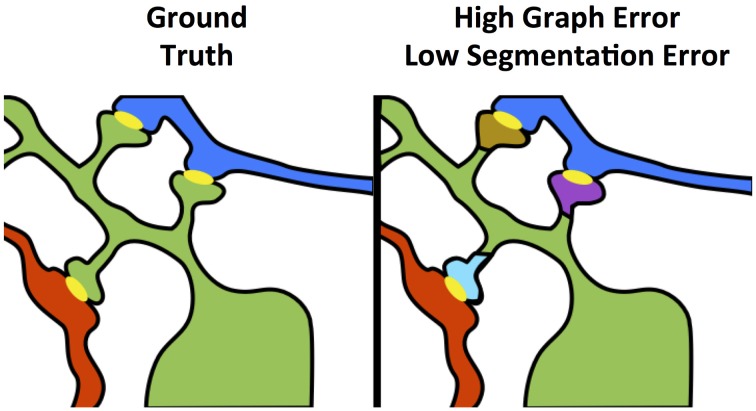
**Yellow objects represent synaptic junctions; other colors are different neurons**. The left panel shows true connectivity; the right panel shows the effect of fragmenting neurons at the dendritic spine necks, which produces a very small change in segmentation error, but a dramatic impact to graph error.

As shown in Figure 4, even small neuron segmentation errors that affect network connections are potentially very significant in terms of the resulting graph, while large errors not affecting topology may be much less significant. These small, significant errors occur frequently on narrow, fragmented spine necks, breaking connectivity between the synapse on the spine head and the parent dendritic shaft (Kaynig et al., 2015).

**Figure 5:**
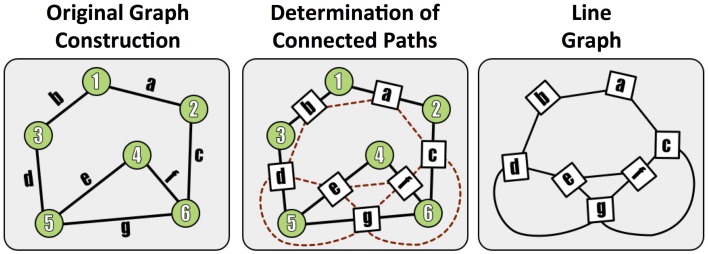
**A demonstration of the methods used to construct a line (edge-based) graph from a conventional node based network, by finding paths between edges in the original graph**.

To assess graph error, we first form the *line graph*, which is the dual of the traditional graph, and represents connections (i.e., paths) between terminals. In this formulation, the synapses become the graph nodes and the graph edges are constructed from the neurons (Figure 5). A non-zero edge *l*_*ij*_ in the line graph represents a path between *synapse i* and *synapse j*.

Line graphs are constructed for both the estimated ℒ{*G*_*estimated*_} and true ℒ{*G*_*true*_} neuronal graphs, resulting in square, undirected, binary matrices. To directly compare the graphs, we augment both matrices so that every node (i.e., synapse) in both graphs has a direct correspondence. We first find common synapses in the detected and true volumes by spatially overlapping annotations. We then augment the graphs with the synapses absent in either graph to create a superset containing all true and detected synapses, leading to true and test graphs of equal size (and corresponding nodes and edges). This graph correspondence is much easier to determine in the line graph (since synapses are small, compact objects) than in the traditional graph formulation (which often contains many neuron fragments).

We propose two graph error metrics; the first is simply the Frobenius norm between the true and test graphs: (Equation 2).

(1) $$G_{err}=||ℒ\{G_{true}^{*}\}-ℒ\{G_{estimated}^{*}\}|{|}_{F}$$

This norm is defined (Golub and Van-Loan, 1996) as:

(2) $$∥A{∥}_{F}=\sqrt{\sum_{i=1}^{m}\sum_{j=1}^{n}|a_{ij}{|}^{2}}$$

This metric is attractive in its simplicity, but has a few major disadvantages. This measure is unbounded, and the error will tend to increase with graph size; it is potentially misleading because it rewards the disproportionately large number of true negative edges in sparse graphs.

The second formulation computes the F_1_ score of the detected edges in the line graph, compared to a truth baseline Section 2.2.1. In this paradigm, true positive edges (TP) are occur in both the true and test graphs; false positive edges (FP) are present in the test graph and not in the true graph, and false negative edges (FN) are true edges missed in the test graph. Similar to an object detection setting, precision, recall, and the F_1_ score are computed for the test graph:

(3) $$Precision=\frac{TP}{TP+FP}$$

(4) $$Recall=\frac{TP}{TP+FN}$$

(5) $$F_{1graph}=\frac{2\times Precision\times Recall}{Precision+Recall}$$

Our metric is interpretable, because true connections are the non-zero common entries. Furthermore, each incorrect entry represents a false positive (spurious connection) or false negative (missed connection). A connection between two synapses in a line graph is equivalent to those synapses being coincident on a neuron. This metric has scalability advantages over voxel-based metrics, because it can be easily computed on large volumes and can be used to characterize common errors. This measure is on [0, 1], and is robust to graph sparseness—true negatives do not impact the metric, and this prevents a class of misleading results (e.g., an empty graph).

For our application (and in this manuscript) we optimize algorithm selection based on graph F_1_ score. Researchers may choose a different optimization goal depending on their application (e.g., maximizing recall with acceptably high precision). A variety of metrics are computed (TP, FP, FN, precision, recall, F_1_, Frobenius norm) for each graph and available online supplement.

#### 2.2.2. Algorithms

Our approach transforms an image volume of cortical tissue into a wiring diagram of the brain. To assemble this pipeline, we begin with membrane detection (Ciresan et al., 2012), and then create three-dimensional neuron segments, using Rhoana (Kaynig et al., 2015), Gala (Nunez-Iglesias et al., 2013), or a watershed-based approach. These are the nodes in our graph, and are compared using the Adjusted Rand index, computed by comparing to neuroanatomist created ground truth, following community convention.

**Figure 6:**
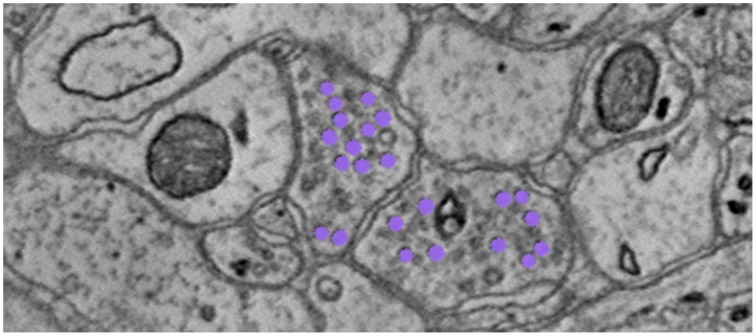
**Depiction of neurotransmitter-containing vesicles**. The presence of synaptic vesicles is the most important feature for our Random Forest, and likely for many manual annotators.

To find the graph edges, we develop a lightweight, scalable Random Forest synapse classifier (Gray Roncal et al., 2015). We combine texture (i.e., intensity measures, image gradient magnitude, local binary patterns, structure tensors) with biological context (i.e., membranes and vesicles). We pruned a large feature set to just ten features and used this information to train a random forest. The pixel-level probabilities produced by the classifier were grouped into objects using size and probability thresholds and a connected component analysis. This method requires substantially fewer computational resources than previous methods (e.g., Becker et al., 2013), which enables large-scale processing. A key insight was identifying neurotransmitter-containing vesicles present near (chemical, mammalian) synapses. These were located using a lightweight template correlation method and clustering (Figure 6). Performance was further enhanced by leveraging the high probability membrane voxels (described above), to restrict our search, improving both speed and precision. Our synapse performance was evaluated using the F_1_ object detection score, computed as the harmonic mean of the precision and recall, based on a gold-standard, neuroanatomist-derived dataset. We took care to define our object detection metric to disallow a single detection counting for multiple true synapses, as that result is ambiguous and allows for a single detection covering the whole volume to produce an F_1_ score of 1 (Gray Roncal et al., 2015).

Synapse and neuron association is completed by finding the neuron labels (i.e., graph nodes) that overlap most frequently with the labeled voxels from each synapse object (i.e., graph edge). This association is recorded via bidirectional linkages in the RAMON objects' metadata fields. Metadata assigned to each object can be traversed server side to construct a graph (Burns et al., 2013), or the graph can be built programmatically at small scales. Output graphs are converted via a web-interface to a community compatible format of choice using MROCP (Mhembere et al., 2013), such as GraphML.

#### 2.2.3. Data

Our experiments utilize a large, publicly available volume of mouse somatosensory (S1) cortex (Kasthuri et al., 2015), imaged using scanning electron microscopy at 3 × 3 × 30 nm per voxel (8-bit data) (Hayworth et al., 2006), aligned and ingested into the Open Connectome Project infrastructure. All images were color corrected (Kazhdan et al., 2015) and downsampled by a factor of two in the imaging plane. The entire raw data volume is approximately 660 GB. The inscribed cube for our deployment workflow is 6000 × 5000 × 1850 voxels (56 GB), or roughly 60,000 um^3^.

#### 2.2.4. Training pipeline

To prepare for algorithm evaluation and testing, we first need to train a variety of algorithms used in the pipeline. For these tasks, we select a data region separate from our evaluation region. Our primary training region was a 1024 × 1024 × 100 voxel region (known to the community as AC4). Gold-standard annotations for both neurons and synapses exist for this volume, based on expert neuroanatomist tracings. Our training tasks include: selecting a template for our vesicle detection module; training our deep-learning membrane classifier on 2D patches; building a random forest classifier for our synapse detection module; and training a Gala agglomerative classifier.

#### 2.2.5. Evaluation pipeline

**Figure 7:**
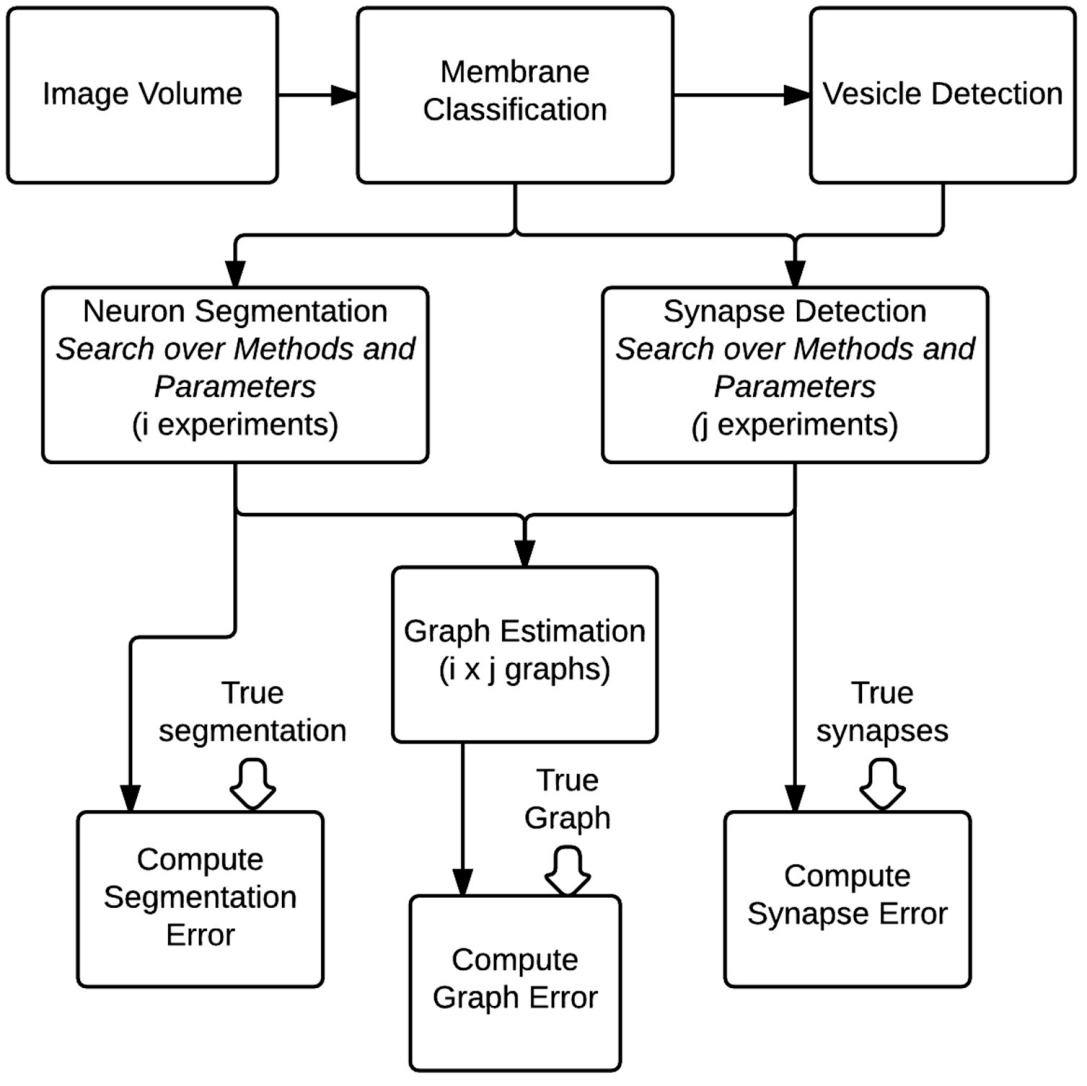
**An overall view of the images-to-graphs Evaluation Pipeline, beginning with image data and ending with graph creation**. Graphs are estimated and evaluated for each combination of i segmentation experiments and j synapse detection experiments.

To evaluate the optimal setting for generating graphs from volumes of brain images, we construct a fully automated pipeline to conduct a hyper-parameter search of different algorithms and their parameters and evaluate them based on community-suggested measures of synapse error, segmentation error, and our novel graph error metric (Figure 7). Other metrics can be straightforwardly added if desired. For evaluation, we use a separate, previously unseen region of 1000 × 1000 × 100 voxels (known to the community as AC3). Gold-standard annotations for both neurons and synapses exist for this volume, based on expert neuroanatomist tracings.

#### 2.2.6. Deployment pipeline

**Figure 8:**
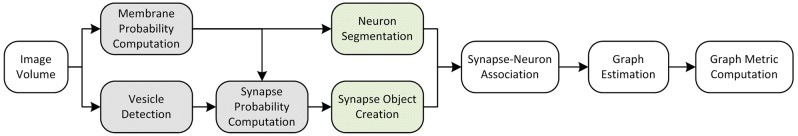
**An overall view of the images-to-graphs Deploy Pipeline, beginning with image data and ending with graph creation**. Modules in white are executed each time, modules that are gray (darkly shaded) are executed once and not varied in our analysis, and modules lightly shaded represent our parameter space.

In this pipeline, we run on a large volume for connectomic analysis (Figure 8). Based on the classifiers created in the training workflows and the operating points found in the evaluation pipeline above, we select an operating point and deploy our end-to-end images-to-graphs pipeline as a reference implementation over a large volume (the entire inscribed dataset).

## 3. Results

The images-to-graphs pipeline allows us to address the question of graph quality and begin to optimize results. We take a systems view of the connectomics problem and evaluate a set of hyper-parameters (i.e., both entire algorithms and parameters within algorithms) to determine the best operating point. In principle, parameters across all modules can be explored; we limited our experiment to variations in neuron segmentation and synapse detection methods for simplicity.

### 3.1. Experiments

We initially performed a parameter sweep to determine the best operating point for our chosen metric, and then applied those parameter settings in a deployed setting.

#### 3.1.1. Evaluation

**Figure 9:**
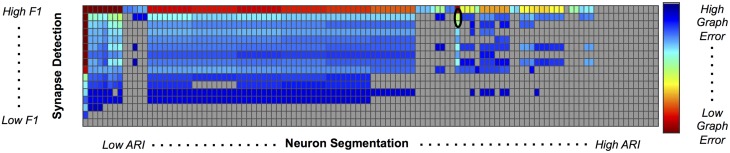
**Experimental Graph Based Error**. 1856 graphs were created by combining 16 synapse detector operating points (rows) with 116 neuron segmentation operating points (columns). The rows are ordered by synapse F_1_ score, and the columns by segmentation adjusted Rand index. The first row and column represent truth, and the upper left corner of the matrix has an error of 0. Cell color reflects graph error (clipped to show dynamic range), with a dark red indicating lowest error and dark blue indicating highest error. Values shaded in gray are not significant; the selected operating point (max F_1_ graph score is circled in black.

We used our pipeline to examine the interaction and settings of the segmentation algorithm and the synapse detector that achieve the optimal graph F_1_ score. Our evaluation varied neuron segmentation parameters (e.g., membrane strength, thresholds, number of segmentation hypotheses). Our synapse operating points were chosen by sweeping over size and probability thresholds. Combinations of these parameters were tested, and the results are displayed as a matrix in Figure 9. We examined 1856 possible graphs, requiring approximately 8000 cluster jobs and over 3 TB of data manipulation. The entire evaluation workflow takes approximately 13 h.

After synapses and neurons were combined to construct a graph, we evaluated the line graph error. A permutation test was run to compute the null distribution of this test statistic. Specifically, we calculate the graph error by uniformly sampling a random graph with the same line graph density as the observed graph for *B* = 10, 000 samples. The *p*-value is then the value of the resulting cumulative distribution function, evaluated at the test-statistic value of the observed graph. We chose a *p*-value significance threshold of less than 0.001; non-significant operating points are shown in gray in Figure 9.

**Figure 10:**
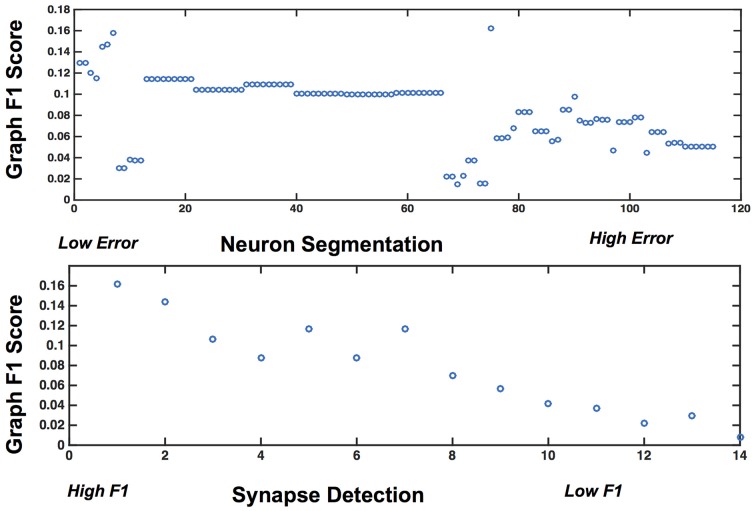
**Plots showing the variability of graph error with segmentation error (top) and synapse error (bottom), for the rows and columns associated with the best operating point**.

Figure 9 shows the results, in sorted synapse and segmentation error order. Each cell in the matrix represents a single graph, and the optimal result is circled in the table. The best result occurs at a segmentation ARI much worse than optimal, and at the maximum synapse F_1_ score. It is clear that constructing the best graph (according to our chosen metric) is more complicated than simply choosing the point with the best synapse F_1_ score and lowest Adjusted Rand index segmentation error. Figure 10 further demonstrates the non-linear relationship between graph error and intermediate measures. By considering the overall problem context, we can select and tune the available algorithms to determine the best result (i.e., operating point) for a given task, such as motif finding. The optimal graph was computed using the Gala segmentation algorithm with an agglomeration threshold of 0.8. The synapse detection probabilities were thresholded at 0.95, and a connected component analysis was used to form the final synapse objects. Objects with a size greater than 5000 pixels in 2D or less than 1000 voxels in 3D were removed to reduce erroneous detections. The optimal graph F_1_ score was 0.16, indicating that significant improvement is needed.

#### 3.1.2. Deployment

The deployment workflow provides a capability demonstration and produced 12,234 neurons with non-zero degree and 11,489 synaptic connections in a volume of ≈60, 000 cubic microns. Total runtime on 100 cores was about 39 h, dominated by the block merging step, which is currently performed serially on each seam. Membrane computation currently takes an additional 3 weeks on our small GPU cluster; this operation is embarassingly parallel and recent advances suggest methods to dramatically speed up this step (Masci et al., 2013).

## 4. Discussion

We have demonstrated the first framework for estimating brain graphs at scale using automated methods. We recast the problem as a graph estimation task and consider algorithm selection and parameter tuning to optimize this objective by leveraging a novel graph error metric. This work provides a scaffolding for researchers to develop and evaluate methods for the overall objective of interest.

We evaluate our pipeline across a set of parameters and modules, leveraging a combination of published methods and novel algorithms. Additional insights may be gained at larger scales and through additional optimization. Although our error metric currently considers only binary, unweighted graphs, there are opportunities to extend this to apply to attributed graphs, as well as to weight the metric by error types (e.g., number of false positives or false negatives).

Automated results do not need to be perfect to draw statistical conclusions, and errorful graphs may be used as the basis for inference and exploitation of “big neuroscience” challenges (Priebe and Sussman, 2012). Bias in errors is another important factor in constructing exploitable graphs that is not fully explored in this manuscript. With the ability to efficiently compare combinations of different algorithms and operating points, we can begin to answer the question of graph quality and how to optimize the results. Having the ability to examine the process from an end-to-end systems approach will enable rapid improvement in graph quality. The infrastructure demonstrated in this work provides a community testbed for further exploration and at-scale computation. Although this manuscript focuses exclusively on electron micrographs, our framework is extensible to many other modalities, including Array Tomography (Micheva and Smith, 2007), CLARITY (Liu and Kao, 2009), and two-photon calcium imaging data.

## Data sharing

All code, algorithms, documentation and data products are open source and released under an Apache 2.0 license. These are available at: i2g.io The brain graphs are produced in both attributed edge and graphml format, and provided to the public for download and analysis. Our analysis routines, such as those included in Figure 9 and Figure 10 are also included in our software repository.

## Author contributions

WGR, DK, RV, MC, and GH designed and conceived of the experiments and provided technical expertise. Graph metrics and statistical analysis were performed by WGR, JV, and CP. WGR, DK, and MP designed and implemented computer vision algorithms. PM, KL, RB, JV, WGR, and DK built the Open Connectome Project and related infrastructure. WGR wrote the manuscript and ran the final experiments, with inputs from all authors.

## Funding

This work is partially supported by JHU Applied Physics Laboratory Internal Research and Development funds and by NIH NIBIB 1RO1EB016411-01.

### Conflict of interest statement

The authors declare that the research was conducted in the absence of any commercial or financial relationships that could be construed as a potential conflict of interest.
